# [Retracted] LncRNA TUG1 serves an important role in hypoxia‑induced myocardial cell injury by regulating the miR‑145‑5p‑Binp3 axis

**DOI:** 10.3892/mmr.2024.13232

**Published:** 2024-04-30

**Authors:** Zhongwei Wu, Shengji Zhao, Chunfu Li, Chaoquan Liu

Mol Med Rep 42: 2422–2430, 2018; DOI: 10.3892/mmr.2017.8116

Following the publication of this article, a concerned reader drew to the Editor's attention that, for several of the figures showing the results of Transwell migration and invasion assay experiments, unexpected areas of similarity were identified in terms of cellular patterns comparing among data panels where the results from differently performed experiments were intended to have been shown, although the areas immediately surrounding these areas often featured comparatively different distributions of cells. Moreover, several of the figures contained invasion/migration assay data that were strikingly similar to data that had appeared in articles published previously by different authors at different research institutes. In addition, the western blots in this article were presented with atypical, unusually shaped and possibly anomalous protein bands in many cases.

After having conducted an internal investigation, the Editor of *Molecular Medicine Reports* has reached the conclusion that the potentially anomalous data in this paper were unlikely to have arisen by coincidence. Therefore, on the grounds of a lack of confidence in the integrity of these data, and given the fact that some of the data were strikingly similar to that which had been published previously in other articles and journals, the Editor has decided that the article should be retracted from the publication. The authors were asked for an explanation to account for these concerns, but the Editorial Office did not receive a reply. The Editor apologizes to the readership for any inconvenience caused, and thanks the concerned reader for drawing this matter to our attention.

